# Clinical Translation of Neutrophil Imaging and Its Role in Cancer

**DOI:** 10.1007/s11307-021-01649-2

**Published:** 2021-10-12

**Authors:** Doreen Lau, Laura M. Lechermann, Ferdia A. Gallagher

**Affiliations:** 1 Department of Radiology, University of Cambridge, Cambridge, UK; 2 Cancer Research UK Cambridge Centre, Cambridge, UK; 3 Present Address: Department of Oncology, University of Oxford, Oxford, UK

**Keywords:** Neutrophils, Cancer, Infection, Inflammation, Imaging, Optical, MRI, SPECT, PET, Immunotherapy

## Abstract

Neutrophils are the first line of defense against pathogens and abnormal cells. They regulate many biological processes such as infections and inflammation. Increasing evidence demonstrated a role for neutrophils in cancer, where different subpopulations have been found to possess both pro- or anti-tumorigenic functions in the tumor microenvironment. In this review, we discuss the phenotypic and functional diversity of neutrophils in cancer, their prognostic significance, and therapeutic relevance in human and preclinical models. Molecular imaging methods are increasingly used to probe neutrophil biology *in vivo*, as well as the cellular changes that occur during tumor progression and over the course of treatment. This review will discuss the role of neutrophil imaging in oncology and the lessons that can be drawn from imaging in infectious diseases and inflammatory disorders. The major factors to be considered when developing imaging techniques and biomarkers for neutrophils in cancer are reviewed. Finally, the potential clinical applications and the limitations of each method are discussed, as well as the challenges for future clinical translation.

**Supplementary Information:**

The online version contains supplementary material available at 10.1007/s11307-021-01649-2.

## Introduction

Neutrophils are granulocytes of myeloid origin characterized by the presence of granules in their cytoplasm and multilobed nuclei. They play critical roles in the innate immunity and are our first line of defense against pathogens such as bacteria and viruses, as well as abnormal cells [1, 2]. Neutrophils regulate many processes, e.g., acute injury and wound repair, autoimmunity, infections, and chronic inflammatory disorders. They are the most abundant immune cells, representing 50–70% of the total circulating leukocytes in humans and 10–30% in mice [3]. It is not clear if this shift towards neutrophil-rich blood in humans has any functional significance [4]. In recent years, a role for neutrophils in cancer has emerged, whereby different subpopulations of neutrophils have been found to possess both pro- or anti-tumorigenic properties.

## Neutrophils: a Double-Edged Sword in Tumor Immunity

**Fig. 1.**
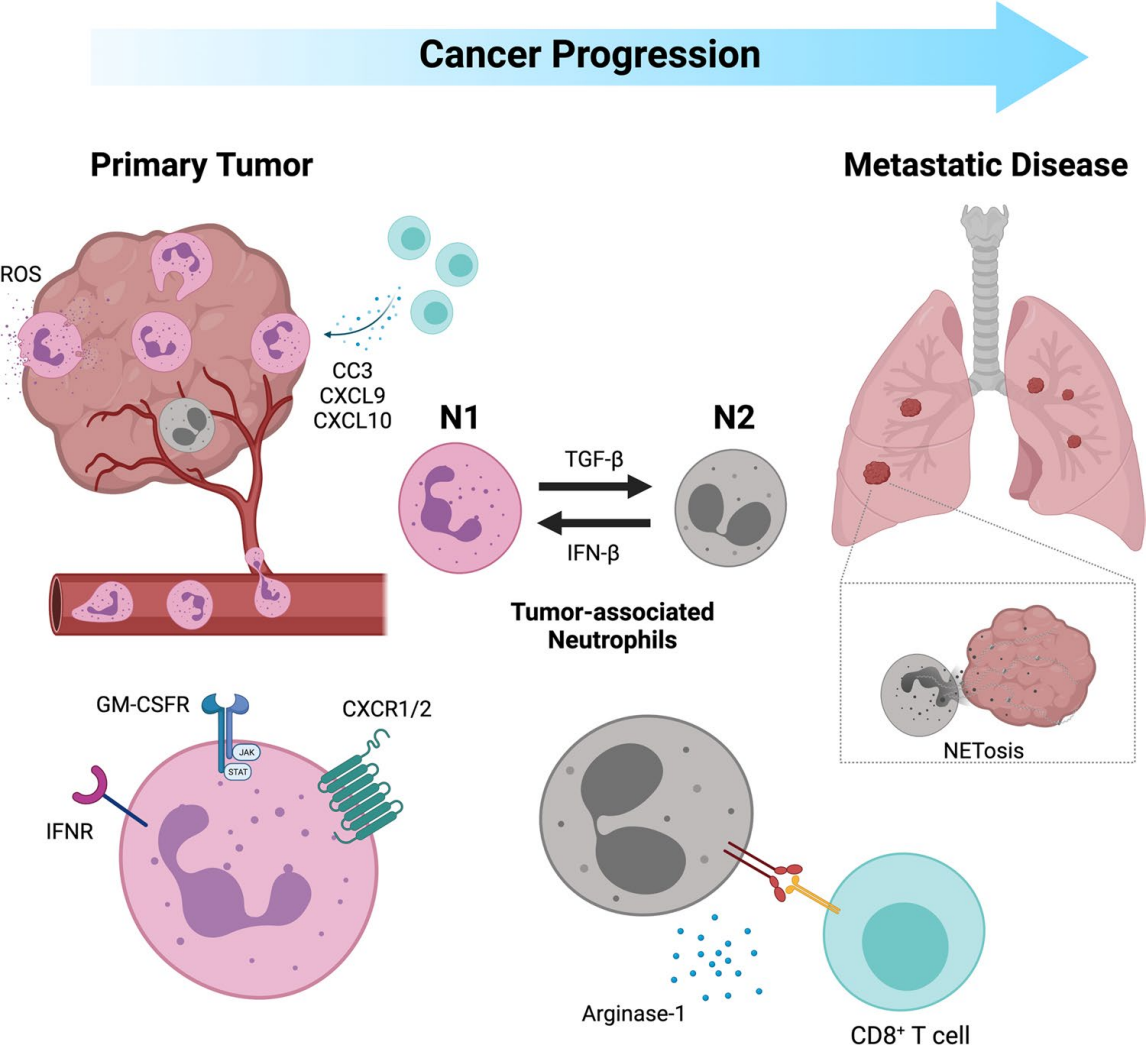
Tumor-associated neutrophils exhibit phenotypic diversity over the course of cancer progression. Oncogenic signaling events which regulate the expression of TGF-β and IFN-β govern the plasticity switch between the anti-tumoral (N1) and pro-tumoral (N2) phenotypes. *Image created with BioRender.com (agreement number MZ22VWEC70)*

Tumor-associated neutrophils (TANs) demonstrate phenotypic diversity during cancer progression and treatment response (Fig. 1). TANs can be classified into two distinct subtypes: N1 and N2 neutrophils. N1 neutrophils are mature high-density cells, which are terminally differentiated and relatively short-lived with a survival time of only 8–12 h in circulation and up to 1–2 days in tissues [1]. N1 neutrophils are anti-tumorigenic, can generate reactive oxygen species (ROS) for cytotoxic killing of tumors, and promote CD8^+^ T cell recruitment and activation through the secretion of T cell chemoattractants such as CCL3, CXCL9, and CXCL10 and proinflammatory cytokines like the granulocyte–macrophage colony-stimulating factor (GM-CSF), tumor necrosis factor alpha (TNF-α), and interleukin 12 (IL-12) [5]. N1 neutrophils express pattern recognition receptors that can detect damage-associated molecular patterns released from abnormal or senescent cells, as well as damaged or necrotic tissues during radiotherapy, to facilitate the clearance of damaged cells [6]. The anti-tumorigenic roles of N1 neutrophils have been widely characterized as an immune defense mechanism during the early stages of primary tumorigenesis.

In comparison, N2 neutrophils possess pro-tumorigenic properties in malignant cancers. They are immature low-density neutrophils, with delayed apoptosis and a prolonged half-life in cancer and inflammatory disorders [1, 7]. Myeloid-derived suppressor cells (MDSCs) have been shown to exhibit similar properties to N2 neutrophils. They promote tumor immunosuppression, proliferation, angiogenesis, and metastasis via the expression of factors such as the programmed cell death receptor ligand 1 (PD-L1), arginase-1, neutrophil elastase (NE), matrix metallopeptidase 9 (MMP9), chemokine receptors (CXCR1 and CXCR2), and vascular endothelial growth factor (VEGF) [8, 9].

TANs exhibit distinct roles and functions in the tumor microenvironment and can be polarized into N1 or N2 subpopulations. This multifaceted identity of TANs is context-dependent and driven by specific inflammatory cytokines and oncogenic processes during cancer progression [8]. Type I interferons (IFN) such as IFN-β, which is constitutively expressed at low levels in many cell types, is important for cancer immunosurveillance [10]. IFN-β has been shown to polarize TANs into the N1 anti-tumor phenotype both in mice and human [11]. In malignant tumors, the expression of the immunosuppressive cytokine transforming growth factor beta (TGF-β) can polarize TANs towards a N2 phenotype. The blockade of TGF-β signaling in syngeneic mouse models of non-small cell lung carcinoma and mesothelioma has been found to promote the cytotoxic and immunostimulatory profile of N1 neutrophils and enhances their recruitment and persistence in the tumor microenvironment via the secretion of neutrophil chemoattractants and expression of adhesion molecules on endothelial cells [12]. Inflammatory cytokines and oncogenic signaling events can influence other immune and stromal cell components in the tumor microenvironment, altering their roles and functions during cancer progression and in turn influencing the phenotypic functions of neutrophils. For example, type I IFNs have known immunostimulatory properties in enhancing dendritic cell maturation, T cell cytotoxicity, survival, and differentiation into memory cells [10]. IFN-γ secreted by type 1 helper T cells (Th1) can induce the polarization of tumor-associated macrophages into an anti-tumor M1 phenotype. These M1 macrophages secrete IFN-β, which in turn can polarize neutrophils to an anti-tumor N1 phenotype. In contrast, the presence of cancer-associated fibroblasts can enhance the recruitment of MDSCs (or “N2 neutrophils”) via the secretion of proinflammatory cytokines such as IL-6, CXCL1, and CXCL2, upregulate the expression of immune checkpoint ligands on neutrophils such as PD-L1, and function as physical barriers to CD8^+^ T cell tumor infiltration [13–15].

As a result of the significant role of TANs in tumor inflammation, various immunotherapeutic drugs have been developed to target TANs in cancer. Some of these treatments are anti-inflammatory drugs repurposed from other immune-mediated diseases to treat cancer by restoring the anti-tumorigenic functions and suppressing the pro-tumorigenic properties of TANs [16, 17].

## Neutrophil Density, Tumor Location, and Prognostic Relevance in Human Cancers and Preclinical Models

### Clinical Studies

Neutrophilia or a high baseline neutrophil-to-lymphocyte ratio (NLR) in the peripheral blood of patients has been associated with poor prognosis in many cancers [18]. A high NLR of more than 5 has been found to be a negative predictor of clinical outcome and response to cancer treatment such as surgery, chemotherapy, and immune checkpoint blockade [19–21]. The mechanisms by which neutrophilia is induced in cancer patients are not fully understood, although aggressive tumors have been shown to secrete granulocyte colony-stimulating factor (G-CSF) at the onset of malignant transformation, resulting in tumor reprogramming of hematopoiesis in the bone marrow and the systemic expansion of circulating neutrophils, which preferentially accumulate in the lungs and facilitate metastatic seeding [22].

The presence and spatial location of TANs have important prognostic implications in cancer [17], and therefore, the localization of TANs within the tumor microenvironment using non-invasive imaging tools could provide important clinical information. For example, infiltration of TANs within the tumor, but not the stroma or tumor periphery, has been associated with poorer prognosis in many cancer types such as non-small cell lung carcinoma (NSCLC), melanoma, and esophageal cancer [17]. However, the opposite has been reported in other cancers such as hepatocellular carcinoma (HCC) and cervical cancer, where a high neutrophil count in the peritumoral and stromal regions, but not intratumorally, has been correlated with poorer objective survival (OS) [23, 24]. These differences in clinical prognosis in relation to TAN location may be due to the spatial heterogeneity in the expression of local environment cues within tumors [17]. For example, in primary colorectal cancer (CRC), TANs have been found to localize predominantly at the invasive margin of tumors and correlated with TGF-β expression [25]. Circulating TANs in stage III-IV CRC have been shown to express higher levels of neutrophil elastase and arginase-1 compared to stage I–II disease, implying a more immunosuppressive phenotype as the disease progresses [26, 27].

### Preclinical Models

The density and function of TANs differ between preclinical mouse models of cancer. The rational selection of the most suitable preclinical model to best represent the human disease for immuno-oncological imaging and therapeutic intervention is crucial [28, 29]. The role of TANs in tumor progression and therapy at the preclinical level is context-dependent and can be influenced by the tumor model and genetic background of the mouse strain used. For instance, differential expression of chemoattractants for neutrophils and MDSCs, such as CXCL1 and CXCL2, has been observed in syngeneic mouse models of carcinogen-induced colorectal tumors, being higher in MC38 tumors compared to the CT26 model [29]. MC38 tumors predominantly comprise polymorphonuclear MDSCs (50.9%) and are not responsive to CTLA-4 and PD-1 therapy, while CT26 tumors have a lower infiltration of MDSCs (20.3%), are rich in cytotoxic immune infiltrates, and are responsive to immune checkpoint inhibitors [29]. The genetic background of the host mouse can also influence neutrophil phenotype and function. For example, comparing two immunocompetent mouse models commonly used in immuno-oncology studies, naïve non-tumor-bearing BALB/c mice contain more peripheral blood neutrophils (10.3% of total immune cells) than C57BL/6 mice (3.3%). The neutrophils of naïve BALB/c mice are of the Ly6G^hi^/CD62L^lo^ phenotype linked to neutrophil aging, whereas the neutrophils in C57BL/6 mice are Ly6G^lo^/CD62L^hi^ and may represent younger neutrophils [28]. Factors such as age- and sex-specific alterations in immune composition may also influence neutrophil function even within the same mouse strain, and therefore, it is important to ensure representative matching of experimental controls when undertaking neutrophil imaging and therapeutic studies [30].

Genetically engineered mouse models (GEMM) which mimic spontaneous and autochthonous tumor growth in human cancers may more closely recapitulate the disease than syngeneic mouse tumors [31]. GEMM tumors arise from key driver mutations in immunocompetent mice. Tumors grow in the presence of intact immune and stromal components, enabling the entire process of cancer progression and tumor immunity to be interrogated. One of the most commonly used GEMMs in immuno-oncology studies is the mouse mammary specific polyomavirus middle T antigen (MMTV-PyMT) model [31]. The overexpression of the oncogene polyomavirus middle T antigen mimics the signaling of receptor tyrosine kinases commonly activated in many human malignancies including breast cancer. The expression of this oncogene in the mammary epithelial cells resulted in the rapid transformation and generation of multifocal breast tumors that readily metastasize to the lungs. CD11b^+^ Ly6G^+^ neutrophils have been found to exist at low levels within the primary tumors of the MMTV-PyMT model and can be systemically mobilized to pre-metastatic niches in the lungs by G-CSF secreted from tumors prior to metastatic colonization [32]. Interestingly, the activation of the C3 complement cascade in a spontaneous small intestinal tumor model (APC^Min/+^ mice) has been found to induce the polarization of TANs to a N2 phenotype and the release of neutrophil extracellular traps (NETs) as the disease progressed. This resulted in blood coagulation and neutrophilia which resemble many human cancers [33]. Pharmacological blockade of CXCR2 in the same model has been demonstrated in another study to attenuate myeloperoxidase MPO^+^ neutrophil recruitment into tumors and suppress the spontaneous formation of benign intestinal lesions [34]. Non-invasive methods to dynamically track neutrophils in human and preclinical models have the potential to reveal novel mechanisms of neutrophil function in cancer, as well as efficiently informing the efficacy of therapeutic intervention.

## Molecular Imaging of Neutrophils at Microscopic and Macroscopic Levels

Immune response and treatment-induced changes in the tumor microenvironment are dynamic processes which require precise monitoring and longitudinal follow-up [35, 36]. Conventional immunological methods like flow cytometry, immunohistochemistry, and other *in vitro* assays are very destructive to tissues and do not provide information on the spatial and temporal heterogeneity of immune response within living organisms which is a hallmark of most tumors and a major driver of therapeutic failure [37].

Molecular imaging methods such as optical imaging, magnetic resonance imaging (MRI), single-photon emission computed tomography (SPECT), and positron emission tomography (PET) have been used for detecting changes in neutrophil function and cellular kinetics in several immune-mediated diseases [38–43]. These imaging methods could also be repurposed for neutrophil imaging in cancer. Neutrophils and associated biomarkers can be imaged at different levels of molecular sensitivity and spatial resolution, from the microscopic observation of single-cell behavior in tissues to the whole-body macroscopic imaging of neutrophil migration and functional status in multiple organs [44] (Supplemental Table S1). A literature search on the use of molecular imaging in examining neutrophil or neutrophil-related biological activity was performed in PubMed including all papers published since the 1970s until July 2021. The search terms used were “neutrophil” AND “imaging” AND “optical” OR “MRI” OR “PET” OR “SPECT”, returning 219 results.

### Optical Imaging

Optical imaging uses light in the visible and near infrared (NIR) range to interrogate cellular and molecular events *in vivo*. Intravital microscopy (IVM) is the most established technique for imaging neutrophil activity at the preclinical level. The method has commonly been used for studying various aspects of innate and adaptive immunity to cancer, infections, and inflammatory disorders. Imaging can be conducted at high spatial and temporal resolution to track individual live cell behavior and cell–cell interactions, and derive metrics such as the velocity, displacement, persistence time, and meandering index from time-lapse videos [15]. Technical advancements in multiphoton microscopy for IVM has enabled live imaging of cells over a longer time frame, as the lower energy lasers of NIR excitation generate lesser cellular damage, photobleaching, and phototoxicity effects on tissues compared to the ultraviolet lasers used in conventional confocal microscopy. The longer wavelength of NIR excitation also allows for deeper imaging of living tissues (up to 1 mm), improves optical sectioning, and minimizes light scattering.

**Fig. 2.**
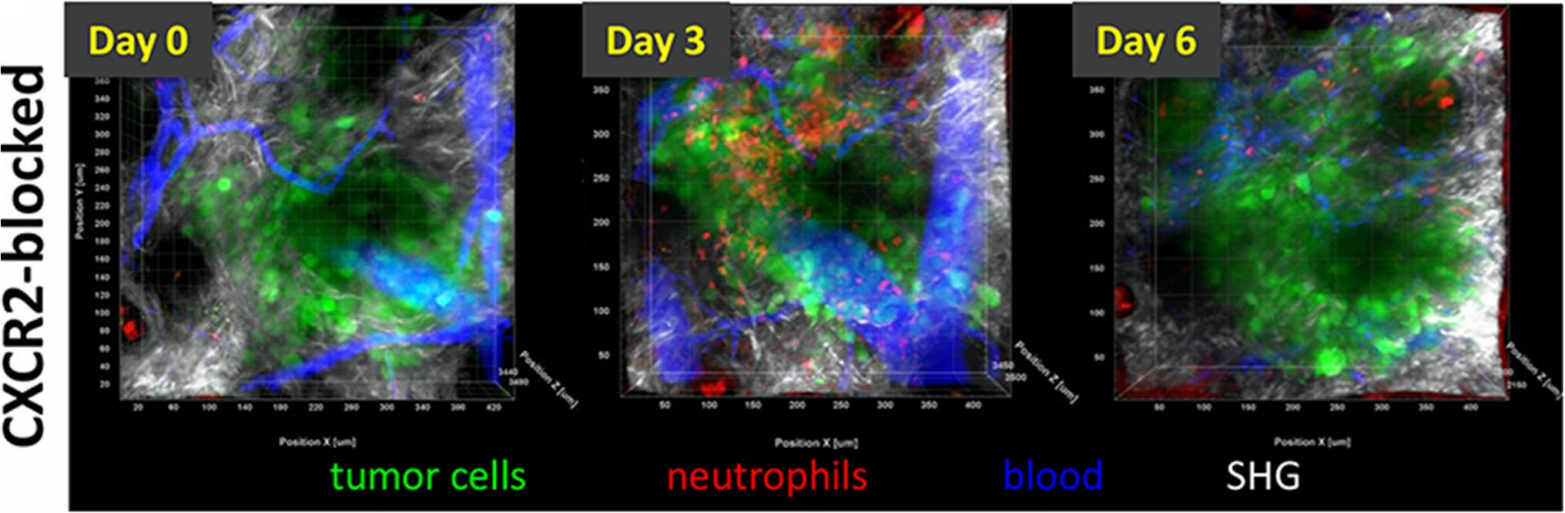
Longitudinal imaging using IVM in Catchup mice implanted with small tumors at the ear pinnae showed early effects of CXCR2 blockade on tumor recruitment of tdTomato^+^ neutrophils on day 0 but not in the later time points at day 3 and day 6 as the disease progressed. *Images reproduced with permission from* [38]

Transgenic mouse models and neutrophil-specific fluorescent probes have been developed for interrogating neutrophil biology *in vivo* using IVM. LysM-EGFP mice have been used for tracking myeloid cells which comprise mostly neutrophils and to a lesser extent monocytes and macrophages [45]. This model has been investigated in breast cancer metastases where GFP^+^ neutrophils were shown to migrate into the lungs facilitating the formation of neutrophil extracellular traps (NETs) for colonization of new metastatic niches [46]. Newer transgenic models like the Catchup mouse, in which the first exon of Ly6G, a granulocyte-specific surface receptor, was replaced by a knock-in allele encoding for the red fluorescent protein tdTomato and Cre recombinase, have been introduced for tracking neutrophil dynamics in cancer and infections [47]. The Catchup mouse has been used for the longitudinal imaging of neutrophil infiltration and persistence in different compartments of small tumor lesions implanted in the mouse ear pinnae [38]. Intratumoral neutrophils demonstrate more persistence and reduced motility compared to peritumoral neutrophils which were observed moving at increasing velocity with tumor progression. The pharmacological blockade of CXCR2 signaling has been shown to inhibit neutrophil trafficking during the early stages of tumorigenesis but not at later time points as the disease progressed (Fig. 2).

Fluorochrome-conjugated antibodies targeting Ly6G and the myeloid differentiation antigen Gr-1 are commonly used for optical imaging of neutrophils in mice. Low doses of injected antibodies (1–40 μg) have been shown to effectively label neutrophils sufficient for several hours of imaging, without causing the depletion of neutrophils or affecting their function and behavior [48]. Although neutrophils can be imaged at microscopic spatial resolution and in multiplex with other cells and tissue structures within the tumor microenvironment using IVM, the method is not easily translatable for clinical use. The technique is often limited by the tissue depth and field-of-view and is invasive when used as a terminal imaging technique on exposed tissues or with implanted window chambers for longitudinal imaging [15, 49].

Nevertheless, clinical translation of optical imaging for studying neutrophil activity in real-time during tissue inflammation may be possible with the development of non-toxic, biologically inert fluorescent probes for optical endomicroscopy (OEM) [50]. For instance, a multi-branched peptide scaffold fluorescent probe, neutrophil activation probe (NAP), has been evaluated in an exploratory trial on clinical OEM of the lungs (*NCT01532024*). This fluorescent probe consists of three internally quenched fluorescein moieties, each conjugated to an optimized peptide sequence that can be phagocytosed by neutrophils and cleaved during the enzymatic process of neutrophil activation as a result of human neutrophil elastase activity. NAP was administered in micro-doses into the distal lungs of healthy volunteers and patients with acute lung inflammation, with no reported adverse reaction. Heterogeneity in contrast uptake was observed in the patients, while no signal was detected in the healthy volunteers [39]. A phase 2 trial is currently underway to evaluate the feasibility of this approach for quantifying neutrophil activation, to predict outcome, and to stratify mechanically ventilated patients in the intensive care unit (*NCT02804854*). However, the application of OEM and other clinical optical imaging techniques for imaging neutrophil activity in cancer on patients is limited by the field-of-view and is rather more suitable for imaging superficially located lesions and tissues with minimal autofluorescence. Molecular imaging techniques such as MRI, SPECT, and PET are more useful for tracking neutrophil dynamics at a whole-body level and across multiple tumors and organs.

### Magnetic Resonance Imaging

MRI is widely used in clinical practice to obtain three-dimensional images at high spatial resolution for diagnosis and treatment monitoring and provides excellent soft tissue imaging using a range of contrast mechanisms. Immune cell tracking in MRI has predominantly been based on superparamagnetic iron oxide (SPIO) nanoparticle labeling of cells and imaging local magnetic field inhomogeneities (T2 and T2* weighting). SPIO-enhanced MRI relies on the phagocytic properties of macrophages, neutrophils, and dendritic cells to efficiently take up SPIOs for magnetic labeling and detection on MRI. Unlike T cells, phagocytes such as neutrophils and macrophages are terminally differentiated, and therefore, the MRI signal from the labeled phagocytes does not decrease due to cell proliferation, although it may be exocytosed into the extracellular space [51, 52]. Ferumoxytol (Feraheme®), an ultrasmall SPIO approved by the US Food and Drug Administration (FDA) for the treatment of iron deficiency in chronic kidney disease, has been used as an off-label MRI contrast agent for the detection of tumor-associated macrophages in patients. In one study, 24 h following intravenous administration of ferumoxytol, a significant correlation was found between tumor T2* signal enhancement (or presence of SPIOs) on clinical MRI and the density of tumor-associated macrophages (both M1 and M2) on histology. The spatial distribution of T2* signal and the localization of macrophages were also found to be different between lymphoma and bone sarcoma [53]. This has demonstrated the feasibility of using ferumoxytol-enhanced MRI as a surrogate imaging biomarker for tumor-associated macrophages in cancer. However, as neutrophils are present in the highest density in human blood (50–70%) compared to macrophages (< 8%), circulating neutrophils may have contributed to the measured SPIO uptake in the tumors, which is a problem that affects many molecular imaging approaches to specifically label neutrophils. Furthermore, the detection of SPIO-labeled cells based on T2* MRI can be compromised by the presence of endogenous iron, hemorrhage, deoxyhemoglobin, and inflammation within the tumor [54]. Marked differences in the pre- and post-contrast images are required to detect differences in SPIO-labeled immune infiltrates. Quantitative measurements of SPIO distributions can also be influenced by factors such as a non-linear relationship between the contrast concentration and T2* relaxation time. Susceptibility artifacts that may arise from air–tissue interfaces, such as in the lung or brain sinuses, can result in an inaccurate estimation of T2* relaxation, especially at very low concentrations of SPIO-labeled cells [55]. Therefore, the use of ferumoxytol-enhanced MRI as a surrogate biomarker for tumor-associated neutrophils requires further validation in future studies.

**Fig. 3.**
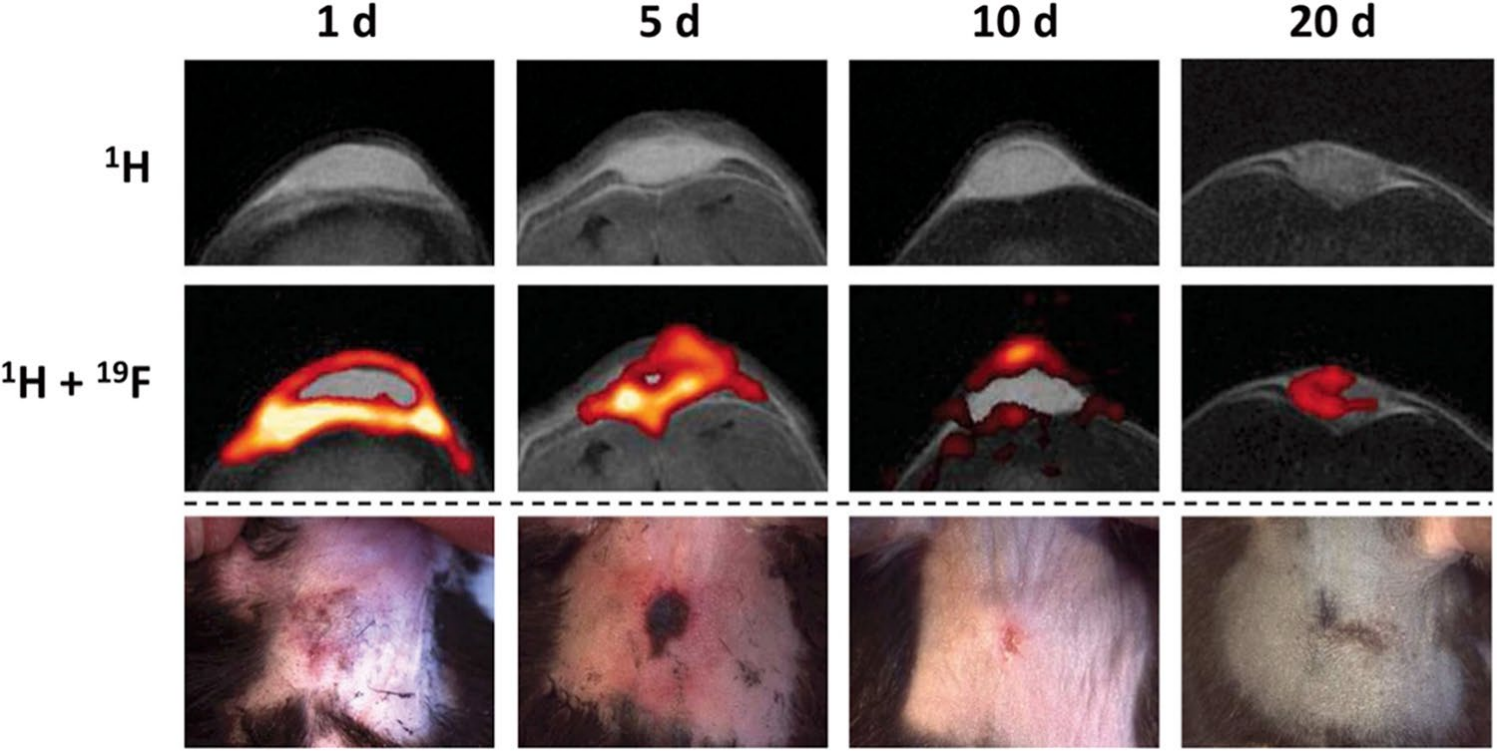
Cellular tracking of changes in ^19^F signal and inflammation resolution over 20 days in an LPS-induced model. *Images reproduced with permission from* [40]

Another approach is fluorine-19 MRI (^19^F-MRI) which may provide a more quantitative method for imaging inflammation. ^19^F has a high relative sensitivity comparable to ^1^H (83% of ^1^H), and the ^19^F signal can be more directly related to the presence of the label, compared to the T2* approaches described above with SPIOs. This quantifiable signal obtained from ^19^F-based contrast agents is highly specific due to the lack of endogenous ^19^F in biological tissues and provides positive rather than negative contrast which facilitates detection [56]. Perfluorocarbons (PFC) have been used for ^19^F-MRI tracking of immune cells both clinically and preclinically. PFCs are biochemically inert, organic molecules that are lipophilic and can be efficiently taken up into cells via passive uptake. PFCs can be emulsified into lipid nanoemulsions or incorporated into the synthetic amino acid poly(lactide-co-glycolide) or PLGA to improve biocompatibility for imaging applications [57, 58]. Commonly used PFCs include perfluoro-15-crown-5 ether and perfluoropolyether [56]. Perfluoro-15-crown-5 ether has been used for imaging neutrophils and monocyte infiltration in a preclinical model of lipopolysaccharide (LPS) bacterial endotoxin-induced inflammation [40]. The magnitude of the ^19^F signal at the inflamed site was shown to correlate with the degree of tissue inflammation and dose of LPS applied. Longitudinal tracking of the entire process of inflammation and its resolution was possible. A gradual decrease in ^19^F signal was detectable on MRI over a period of 20 days (Fig. 3). However, perfluoro-15-crown-5 ether has an extremely long biological half-life. It can accumulate in the liver and spleen and can persist in different organs for several months [59]. This precluded its future clinical application for imaging inflammation and early treatment response assessment. As such, alternative PFCs with high fluorine content and shorter biological half-lives have been tested, in which perfluorooctyl bromide, which has been used in clinical trials as a blood substituent, demonstrated similarly high sensitivity as perfluoro-15-crown-5 ether but better excretion profile (12 days) for *in vivo* imaging [56, 59]. Although SPIOs and ^19^F-PFCs labeling of cells offer the opportunity to track neutrophils without the use of ionizing radiation, MRI and MR spectroscopy is often limited by the poor sensitivity and significant concentration of contrast agents required for detection compared to nuclear medicine imaging.

### Radionuclide Imaging

Gallium-67 (^67^Ga)-labeled citrate has been used for over 30 years in SPECT imaging of infections and inflammatory disorders. Gallium functions as an iron analogue *in vivo* by binding to circulating transferrin and extravasates within inflammatory sites due to local increase in blood flow and vascular permeability [60]. The exact mechanism for tissue retention and neutrophil uptake of the [^67^Ga]Ga-citrate/transferrin complex is unclear but has been postulated to be either due to gallium exchange with iron-binding proteins such as lactoferrin released by neutrophils at the inflamed site or due to phagocytic uptake of bacteria containing siderophores bound to gallium [60, 61]. The long half-life of gallium-67 (*t*_1/2_ = 78.3 h) can potentially be replaced with gallium-68 (*t*_1/2_ = 68 min) which has a suitable half-life and better sensitivity on PET imaging for routine clinical application [62]. Furthermore, the kinetics for chemotaxis of innate immune cells is usually fast, and neutrophils are known to be recruited to the site of inflammation within minutes [2]. Although [^67^Ga]Ga-citrate has demonstrated good sensitivity in detecting a variety of acute and chronic infections and inflammatory disorders, the technique is more challenging for imaging inflammatory processes in cancer due to the non-specific accumulation of [^67^Ga]Ga-citrate in malignant tissues, e.g., due to enhanced permeability and retention (EPR) effects [63]. [^67^Ga]Ga-citrate also exhibits non-specific intra-abdominal activity due to slow excretion via the gastrointestinal tract and uptake in the liver and skeleton. Furthermore, the radiopharmaceutical can be retained in normal tissues such as the nasopharynx, breast, lacrimal and salivary glands due to the presence of lactoferrin. These challenges have hampered the application of [^67^Ga]Ga-citrate for imaging tumor inflammation in metastatic cancers [64].

White blood cell (WBC) scintigraphy using *ex vivo* radiolabeled leukocytes and planar scintigraphy is a specific gold standard technique that has been used routinely in clinical practice for detecting unknown sites of infection and inflammation. Although this involves labeling the full range of circulating white blood cells in the peripheral blood, the majority of these are neutrophils. Since its introduction in 1976, WBC scintigraphy has been used in various clinical settings for the diagnosis of diseases such as vascular graft infections, inflammatory bowel disease (IBD), and osteomyelitis [65]. In recent years, radiolabeled autologous neutrophils and SPECT imaging have been used in experimental medicine studies on human for investigating changes in neutrophil kinetics in inflammatory disorders such as acute respiratory distress syndrome (ARDS) and chronic obstructive pulmonary disorders (COPD), as well as in primary lung cancer [41, 66, 67].

Technetium-99 m (^99m^Tc) hexamethylpropyleneamine oxime (HMPAO) and indium-111 (^111^In) oxine are the most common radiopharmaceutical agents used for the direct labeling of human neutrophils [65]. Neutrophils are ideal leukocytes for direct cell labeling using radiopharmaceuticals as gamma irradiation at doses less than 175 Gy has not been shown to cause alterations in neutrophil migration, ROS production, cytotoxic killing, or phagocytosis [68]. The choice of radiopharmaceutical for specific labeling and *in vivo* tracking of neutrophils depends on the type of inflammation (acute versus chronic) and the circulating half-life and life span of neutrophils in the disease of interest. For example, technetium-99m has a short half-life (*t*_1/2_ = 6 h) sufficient for imaging acute inflammation which usually involves mature neutrophils with a shorter circulating time. Indium-111 has a relatively long half-life (*t*_1/2_ = 2.8 days) useful for detecting chronic inflammation in peripheral tissues including tumors containing neutrophils with heterogenous mature and immature phenotypes and a prolonged circulating time [1, 69]. The size of the circulating and marginated pools of neutrophils can be altered in cancer and other immune-mediated diseases [2], and the dynamics of radiolabeled neutrophils can be tracked non-invasively using imaging.

SPECT imaging of [^111^In]In-tropolone radiolabeled neutrophils has been investigated as an approach for studying neutrophil kinetics in four patients with primary lung cancer prior to surgical resection [41]. Intratumoral uptake of radiolabeled neutrophils was detected in a patient with squamous cell carcinoma at the 20-h scan. The gamma counts of tissue cores taken from different sites of the tumors from all patients were found to correlate positively with myeloperoxidase MPO^+^ neutrophils detected on histology. No obvious tumor uptake in neutrophils on gamma counting nor histology was found in a patient with lung adenocarcinoma. Interestingly, MPO^+^ neutrophils were also detected on histology in tumor regions where the gamma counts were low, implying the presence of tissue-resident neutrophils that were not accounted for when imaging radiolabeled neutrophils isolated from the peripheral blood. However, the pro- or anti-tumoral phenotype and activation status of the migrating neutrophils cannot be discerned from tracking these labeled cells. Furthermore, the procedures involved in direct cell labeling, i.e., isolation, purification, and radiolabeling of cells, are relatively time-consuming and would usually take 2–3 h before the radiolabeled neutrophils can be reinjected back into the same patient for imaging [65].

The development of radiopharmaceuticals based on antibodies, peptides, and small molecules which can be directly injected into patients and bind to neutrophils *in vivo* would provide complementary and additional information to direct cell labeling approaches. Several radiopharmaceuticals have been developed for imaging neutrophils in infection and inflammatory disorders, some of which have been used clinically and can potentially be translated for cancer diagnosis and treatment evaluation. Monoclonal antibodies targeting cell surface glycoproteins expressed on human neutrophils and granulocytes have been evaluated in clinics. One of the very first examples is [^99m^Tc]Tc-fanolesomab (NeutroSpec™, Palatin Technologies, USA), a murine IgM anti-stage-specific embryonic antigen-1 (SSEA-1) targeting the glycoprotein CD15 expressed on activated human neutrophils [70]. ^99m^Tc-fanolesomab has demonstrated high binding affinity to activated neutrophils (*K*_d_ = 10^−11^ mol/L) without affecting their chemotactic behavior. The tracer exhibited rapid clearance from the circulation due to specific binding to circulating neutrophils and had comparable diagnostic accuracy to WBC scintigraphy. [^99m^Tc]Tc-fanolesomab was approved in 2004 by the US FDA for the diagnosis of appendicitis and osteomyelitis [71]. It was available in a kit formulation for rapid labeling with [^99m^Tc]Tc-pertechnetate in a simple 5-min procedure, avoiding the time-consuming steps associated with WBC labeling. However, transient neutropenia was reported after its introduction due to specific neutrophil binding and sequestration of the tracer in the liver. Unfortunately, [^99m^Tc]Tc-fanolesomab was suspended from the market in 2005 due to reports of fatal cardiopulmonary reactions associated with its use, the cause of which is unknown and the future of [^99m^Tc]Tc-fanolesomab remains uncertain.

Although there have not been other tracers clinically available for detecting CD15 or neutrophil-specific cell surface marker, monoclonal antibodies and antibody fragments targeting the surface antigens on granulocytes (majority neutrophils) have been developed for imaging inflammation and infection. A notable example is [^99m^Tc]Tc-sulesomab (LeukoScan™, Immunomedics GmbH, Germany), a murine antibody fragment (Fab’) targeting the non-specific cross-reacting antigen-90 (NCA-90) or CD66c, a cell adhesion molecule expressed on activated granulocytes including neutrophils [72]. [^99m^Tc]Tc-sulesomab has been licensed for several years in Europe for imaging musculoskeletal infections. The tracer has demonstrated increased binding to granulocytes *in vitro* following priming and activation. The uptake of [^99m^Tc]Tc-sulesomab in patients with musculoskeletal infections has been shown to be due to local binding to activated granulocytes and increased vascular permeability in inflamed lesions. The tracer exhibited a relatively good blood clearance profile, minimizing the background blood signal [73]. As [^99m^Tc]Tc-sulesomab is a Fab’ fragment, it does not induce significant immunogenicity against murine antibodies compared to the use of full-length antibody equivalent [72]. Hence, [^99m^Tc]Tc-sulesomab is relatively safe for use in follow-up scans and tracer re-administration in therapeutic studies. It may be feasible as a clinically available tool for imaging granulocytic infiltration and as a surrogate biomarker for neutrophils in cancer.

**Fig. 4.**
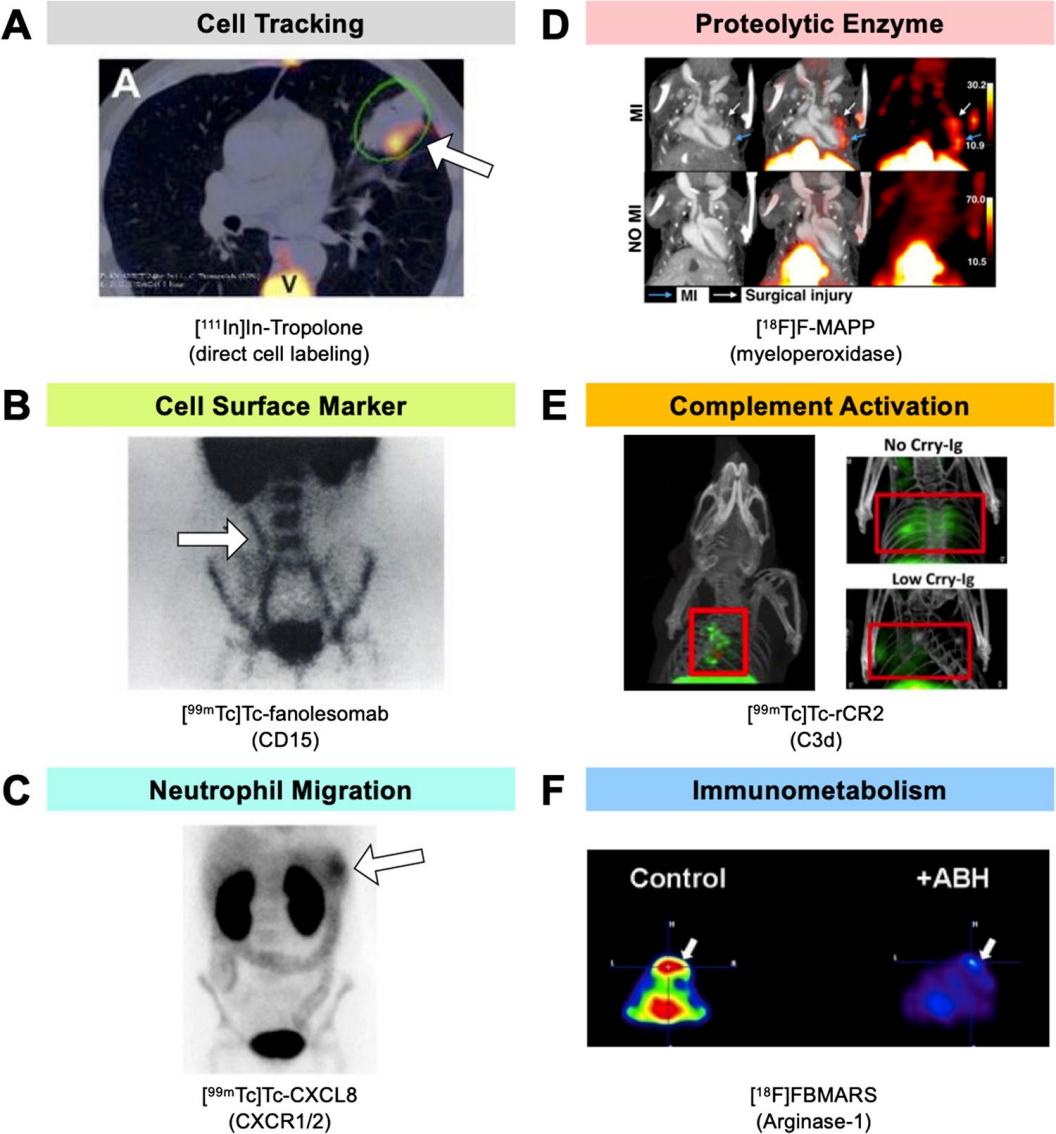
The cellular kinetics and molecular events involved in neutrophil function in cancer can be imaged using radiopharmaceuticals developed originally for imaging infection and inflammatory disorders. **A** Neutrophils can be directly labeled with lipophilic radiopharmaceuticals such as [^111^In]In-tropolone for cellular tracking *in vivo* [41]. **B** Non-invasive detection of neutrophils based on the expression of its specific cell surface antigen activation marker CD15 has been performed clinically using [^99m^Tc]Tc-fanolesomab [70]. **C** Neutrophil migration to sites of inflammation has been studied using the radiolabeled chemokine [^99m^Tc]Tc-CXCL8 [42]. **D** Activation of neutrophils in inflamed tissues can be visualized non-invasively by imaging the activity of proteolytic enzymes such as myeloperoxidase with the small molecule [^18^F]F-MAPP [43]. **E** The role of complement activation in the inflammatory processes of neutrophil recruitment and activation and dose-dependent changes with treatment can be probed with a radiolabeled natural ligand of C3d complement receptor-2, [^99m^Tc]Tc-rCR2 [88]. **F** The involvement of neutrophils in immunometabolism and tumor metabolic immunosuppression can be imaged using a radiofluorinated small molecule inhibitors of arginase-1, [^18^F]FBMARS [101]. *Images reproduced with permission from* [41–43, 70, 88], and [101]

Other than cell surface markers expressed on neutrophils, newer radiopharmaceuticals have been developed for visualizing various molecular events involved in neutrophil function. These include tracers targeting the chemokine signaling pathways, proteolytic enzymes involved in neutrophil antiviral and antimicrobial functions, and complement cascade in neutrophil activation, as well as immunometabolism associated with neutrophils (Fig. 4 and Table 1). These biological processes are important pharmaceutical targets for cancer and are potential imaging biomarkers for disease prognosis and treatment stratification [16].

**Table 1.** Radionuclide imaging approaches to probe the molecular events associated with neutrophil activity in cancer, infections, and inflammatory disorders

Biological target	Radiopharmaceutical	Application	Stage of testing	Reference
**Neutrophil migration**				
CXC chemokine receptor CXCR1 and 2	[^99m^Tc]Tc-CXCL8	Colitis Inflammatory bowel disease	Preclinical Clinical	[76] [42]
**Proteolytic enzymes**				
Myeloperoxidase	[^18^F]F-MAPP	Myocardial infarction	Preclinical	[43]
Neutrophil elastase	[^99m^Tc]Tc-MAG_3_-EPI-HNE-2	Bacterial infection	Preclinical	[77]
**Complement activation**				
Complement component C3a	[^99m^Tc]Tc-rCR2	Ischemia–reperfusion injury	Preclinical	[87, 88]
**Immunometabolism**				
Arginase-1	[^18^F]FBMARS	Asthma and prostate cancer	Preclinical	[101]

#### Neutrophil Migration

TANs and MDSCs migrate towards a concentration gradient of chemokines secreted from tumors and metastatic niches via the expression of chemokine receptors CXCR1 and CXCR2 [74]. Elevated serum IL-8 levels (also known as CXCL8) have been associated with a higher density of TANs (predominantly N2) and poorer response to immune checkpoint inhibitors in patients with advanced cancers [75]. As such, several drugs targeting CXCR1 and CXCR2 are currently in clinical trial for treating cancer such as metastatic melanoma (*NCT03161431*).

[^99m^Tc]Tc-CXCL8 has been synthesized for detecting CXCR1- and CXCR2-mediated neutrophil recruitment in several immune-mediated diseases in preclinical models and patients [42, 76]. Inflammatory lesions of experimental colitis in rabbits were detected within 1 h following administration of [^99m^Tc]Tc-CXCL8 and was superior to [^99m^Tc]Tc-HMPAO-labeled granulocytes [76]. In a prospective trial conducted on 30 patients with IBD, [^99m^Tc]Tc-CXCL8 was able to detect active disease with an overall sensitivity and specificity of 95% and 44% compared to 71% and 70% for endoscopy [42]. The accumulation of [^99m^Tc]Tc-CXCL8 correlated to the degree of neutrophil infiltration in the affected mucosa on histology. However, in some cases, increased uptake of [^99m^Tc]Tc-CXCL8 was observed despite the absence of inflammatory features detected on endoscopy. This was postulated to be due to the difference in penetration depth limit between whole-body imaging and optical imaging, as the center of inflammation might be deeper than the mucosa (transmural involvement) and thus not detectable by endoscopy. This demonstrated potential in using [^99m^Tc]Tc-CXCL8 for detecting neutrophil recruitment in cancer and therapeutic response. However, when using [^99m^Tc]Tc-CXCL8 to evaluate the treatment effects of CXCR1/2 inhibitors, it is important to understand the pharmacokinetics and clearance rate of CXCR1/2 inhibitors and determine a suitable imaging time point for treatment follow-up assessment. This would avoid detection of false negatives due to the blocking effects of drugs that may bind in a similar fashion as the same epitope of the tracer.

#### Proteolytic Enzymes

Several radiopharmaceuticals have been developed for imaging the proteolytic activity of enzymes involved in the antiviral and antimicrobial functions of neutrophils [43, 77]. A fluorine-18 radiolabeled myeloperoxidase-activatable PET probe ([^18^F]F-MAPP) has been synthesized for detecting MPO activity *in vivo* [43]. MPO is predominantly expressed in the azurophilic granules of neutrophils and released during phagocytosis to generate reactive oxygen species for neutrophil cytotoxic killing of pathogens or abnormal cells [78]. Tumor infiltration by MPO^+^ neutrophils has been associated with a favorable prognosis in colorectal and breast cancers [79, 80]. [^18^F]F-MAPP was shown to accumulate in mice at Matrigel sites implanted with human MPO. Specific uptake of [^18^F]F-MAPP by four-fold was detected in the affected tissues of wild-type mice with myocardial infarction. No uptake was seen in MPO-deficient mice with myocardial infarction. These results demonstrated the potential for using [^18^F]F-MAPP for the non-invasive detection of MPO activity. Furthermore, [^18^F]F-MAPP has a shorter radioactive half-life and molecular size compared to other ^111^In- and ^67^Ga-based SPECT tracers for MPO detection, which would be more ideal for clinical translation [81, 82].

Neutrophil elastase (NE) is another attractive target for pharmaceutical intervention and imaging due to its implications in neutrophil-mediated cancer metastasis [83]. A radiolabeled peptide [^99m^Tc]Tc-MAG_3_-EPI-HNE-2 based on a DNA aptamer capable of inhibiting human NE has been developed for imaging NE in infected non-human primates [77]. [^99m^Tc]Tc-MAG_3_-EPI-HNE-2 accumulated rapidly in sites of bacterial infection following administration. However, due to its low molecular weight, [^99m^Tc]Tc-MAG_3_-EPI-HNE-2 exhibited rapid blood clearance and high non-specific uptake in the kidneys. This masked the detection of infected lesions near the kidneys and limited the clinical applicability of this approach.

#### Complement Activation

The activation of the complement components C3 and C5 has been known to activate and recruit neutrophils to sites of complement activation for cytotoxic killing of tumor and clearing of dead cells via phagocytosis in treatment response [84]. Generation of the cleavage product C3d following C3 activation has been associated with N1 neutrophil recruitment to damaged or stressed cells following radiotherapy, as well as in other forms of tissue injury and infection [85, 86]. A radiolabeled peptide based on the natural ligand of C3d, complement receptor-2 [^99m^Tc]Tc-rCR2, has been developed for the non-invasive imaging of complement activation following ischemia–reperfusion injury (IRI) in a murine model of cardiac transplantation [87]. Higher uptake of [^99m^Tc]Tc-rCR2 was detected in the heart isografts following IRI in wild-type mice and confirmed on *ex vivo* biodistribution studies and histology. No accumulation of tracer was observed in the isografts in C3^−/−^ mice following IRI. A dose-dependent reduction in accumulation of [^99m^Tc]Tc-rCR2 was also detected in myocardial IRI mice given low versus high dose of Crry-Ig, an inhibitor of C3 complement activation [88]. Nevertheless, high uptake and non-specific retention of the tracer were seen in the kidneys and bladder. This may limit the clinical translation of [^99m^Tc]Tc-rCR2 for imaging complement activation in tissues or tumors located in the lower abdomen.

#### Immunometabolism

Cellular metabolism plays an important role in governing the function and phenotypic plasticity of many immune cells [89]. Neutrophils have traditionally been thought to be highly dependent on glucose metabolism. However, emerging evidence has demonstrated that neutrophils can upregulate different metabolic pathways such as the tricarboxylic (TCA) cycle, oxidative phosphorylation (OXPHOS), pentose phosphate pathway (PPP), and fatty acid oxidation (FAO) to fulfill their energetic demands and cellular functions. Neutrophils are exposed to a variety of metabolic fuels as they transit through the blood and across different organs and sites of infection, inflammation, and malignant tissues, and this may partly explain their metabolic plasticity [90].

Neutrophils differentially express the glucose transporters GLUT1, GLUT3, and GLUT4 under resting and activated states. They exhibit a rapid increase in glucose consumption following activation [91]. [^18^F]fluorodeoxyglucose ([^18^F]FDG), a radiolabeled glucose analogue, is the workhorse of molecular imaging in cancer. Since its introduction in 1978, the technique has been widely used for cancer detection, staging, and treatment evaluation in patients, as well as for imaging non-specific inflammation and infection in non-malignant tissues [92]. Following intravenous injection, [^18^F]FDG is taken up by glucose transporters, underwent phosphorylation by hexokinase and remained trapped intracellularly [92]. It has been used more specifically for the evaluation of neutrophilic inflammation in canine models of ARDS [93]. [^18^F]FDG uptake was shown to increase in the lungs of endotoxin-treated animals and correlated to neutrophil activation and ^3^H-deoxyglucose consumption in neutrophils obtained from bronchoalveolar lavage [93]. [^18^F]FDG has also been used for detecting immune-related adverse events (irAEs) triggered by the autoimmune side effects of immune checkpoint inhibitors [94]. For instance, persistent colonic wall thickening and surrounding inflammation in the pericolonic fat stranding with intense inflammatory FDG uptake have been reported in metastatic melanoma patients diagnosed with autoimmune colitis following ipilimumab therapy, which subsided after intervention with corticosteroids [94]. However, it is difficult to reliably differentiate inflammatory processes from malignancy as both tumor cells and infiltrating leukocytes (including neutrophils) are metabolically active cell types, and the relative contribution of each cell types to the detected PET signal cannot be deconvolved [95, 96]. Visualization of tumors and inflammatory processes in the prostate and brain are also limited due to high background physiological uptake by the surrounding tissues [97–99].

Amino acid metabolism also plays an important role in regulating tumor immunity. Increased *L*-arginine metabolism by arginase-1 released from TANs (predominantly N2 neutrophils) in the tumor microenvironment has been associated with T cell immunosuppression in mice and humans [100]. Radiopharmaceuticals based on arginase-1 inhibitors have been developed for imaging arginase expression in preclinical models of asthma and prostate cancer [101]. Specific uptake of the radiolabeled arginase inhibitor [^18^F]FBMARS was observed on autoradiography of lung sections from a guinea pig model of asthma overexpressing arginase. A significant decrease in tracer accumulation in PC3 tumor xenografts was also detected in mice treated with arginase inhibitors. As [^18^F]FBMARS exhibits a short half-life and fast clearance, it would be ideal for clinical translation as an indirect marker of N2 neutrophils and could potentially be used as a tool to stratify patients for treatment with arginase inhibitors.

## Discussion and Conclusions

The role which neutrophils play in tumor progression and response to therapy is an emerging research area that offers potential new therapeutic targets. For example, the phenotypic diversity and plasticity of neutrophils can be exploited at different stages of tumor growth and response to therapy, whereby neutrophils can be modulated from a pro-tumorigenic to an anti-tumorigenic phenotype. Careful selection of suitable preclinical models with appropriate genetic manipulation and therapeutic interventions is important to recapitulate the human disease, allowing changes in this cell population to be accurately characterized. Biomarkers of neutrophil function derived from whole blood sampling and tumor biopsy are invasive and may not reflect the spatiotemporal dynamics of neutrophil behavior in cancer patients or changes in response to immunomodulatory therapies. The development of non-invasive imaging biomarkers will be crucial for examining the dynamics of neutrophil behavior and function in cancer as well as determining the window of opportunity for therapeutic intervention.

Repurposing of molecular imaging tools already used in other immune-mediated diseases for oncological applications can potentially fast-track the process of examining human neutrophils in cancer, without the need to overcome significant regulatory hurdles associated with the clinical translation of new methods. Non-invasive tracking of neutrophils based on a direct *ex vivo* cell labeling approach can specifically show the distribution of the injected labeled population and how it traffics into the tumor or organ of interest, with little or no background signal to confound the analysis. However, these labor-intensive methods may not be suitable for routine clinical application and for frequent treatment follow-up scans. Moreover, direct cell labeling reveals the presence of neutrophils in tumors but not their functional status. The use of radiolabeled antibodies, peptides, or small molecules for detecting neutrophil-specific biological processes is more clinically manageable and can provide functional information on the cellular activation status or immunosuppression that may reveal the “real identity” of neutrophils. However, depending on the specific biological activity, size, or molecular weight of an imaging probe, it may label more than one resident cell population and could demonstrate non-specific background accumulation which may reduce the specificity and sensitivity for detecting the cell population of interest. When using antibodies to target a specific cell population, it is important to validate their potential antagonistic or agonistic effects on cell function, viability, and proliferation, both *in vitro* and *in vivo* [47]. The depletion of target cells by the presence of an intact Fc region in certain clones of antibodies can be overcome by engineering antibodies with cleaved Fc region or antibody fragments without the Fc receptor [47, 49].

Many imaging modalities have been used to probe neutrophil distribution. SPECT has been used routinely for white cell scintigraphy for many years and more recently has been used to detect enriched and labeled neutrophil populations. PET offers much higher sensitivity than both SPECT and MRI for examining neutrophil biology. The use of short half-life positron-emitting radionuclides in PET enables shorter imaging time points and treatment follow-up scans suitable for a routine clinical workflow and early treatment evaluation. On the preclinical level, IVM represents a highly specific and established method for multiplexing and examining single-cell behavior and cell–cell interactions. However, as this is an invasive method, it is not suitable for longitudinal imaging of immune response. Combining these methods using hybrid imaging may offer significant advantages by exploiting the distinct benefits of each modality. For instance, [^18^F]FDG has been used as part of a hybrid PET/MRI approach to examine musculoskeletal inflammation, providing multiparametric information on the tissue structure, function, and immunometabolic activity in a single imaging session [102, 103].

An important mechanistic approach in the future will be to conduct multiplex *in vivo* imaging of neutrophils with other tumor or immune cell markers such as T cells and PD-L1, using multi-modal or dual-probe imaging approaches, to examine the spatiotemporal dynamics of tumor immunity and treatment-induced changes. Dual-isotope SPECT/CT imaging combining [^99m^Tc]Tc-hydroxymethylene diphosphonate (assessing bone matrix turnover) or [^99m^Tc]Tc-sulfur colloid (measuring bone marrow uptake) with [^111^In]In-leukocytes as measure of inflammation has also been used for diagnosing and localizing musculoskeletal infections with relatively good sensitivity and accuracy [104, 105]. However, dual-isotope imaging is logistically more difficult to perform as part of routine imaging due to the extended imaging time, increased cost, and radiation dose [105]. Although the technique is not suitable for standard of care clinical applications, it is potentially useful for examining the complexity and dynamics of tumor-immune interactions at a whole-body level in experimental medicine studies.

In conclusion, the development and clinical translation of molecular imaging tools and neutrophil imaging biomarkers could provide important diagnostic and prognostic information and address some of the outstanding questions in the field of immuno-oncology. These tools could be used for non-invasive tracking of neutrophil migration and assessment of their retention in tissue and to identify how neutrophil behavior differs from other immune cell subpopulations within the tumor. An improved understanding of how neutrophils interact with other tumor-immune cells in time and space could facilitate the development of new immunotherapies in the future. Important unanswered research questions include the specificity of these labels for neutrophils and the functional effects of the label on the cell. Molecular imaging tools could be used in the future to determine the timing of the plasticity switch between N1 and N2 neutrophils within the tumor, which is a key step for therapeutic intervention. Translating these imaging methods into clinical care is challenging but has the potential to provide important tools for predicting and determining successful treatment response to both conventional and novel therapies.

In conclusion, neutrophils play a significant role in cancer prognosis and response to treatment. With the increasing role of immunomodulatory drugs in cancer care, the development of molecular imaging methods for non-invasive visualization and quantification of neutrophils could provide very powerful clinical decision-making tools as well as companion diagnostics for drug development.

## Supplementary Information

Below is the link to the electronic supplementary material.

Supplementary file1 (DOCX 16 KB)
