# Supplementary material for: Clinical Translation of Neutrophil Imaging and Its Role in Cancer
Source: Mol Imaging Biol. 2021 Oct 12;24(2):221–34. doi: 10.1007/s11307-021-01649-2 (PMC8983506; doi:10.1007/s11307-021-01649-2)
Supplement: Supplementary file 1 — Supplementary file1 (DOCX 16 KB) [file 11307_2021_1649_MOESM1_ESM.docx]

**Supplemental Table S1.** Comparison of molecular imaging modalities used in imaging neutrophils in cancer, infections, and inflammation. Each modality presents advantages and limitations in neutrophil detection and treatment response assessment.

| **Imaging Modality** | **Energy** | **Spatial Resolution** | **Penetration Depth** | **Advantages** | **Limitations** | **Clinically Translatable?** |
| --- | --- | --- | --- | --- | --- | --- |
| IVM | Visible to infrared light | ≥ 1 mm | ≤ 1 cm | - High sensitivity and specificity - Multiplex imaging - Real-time imaging | - Poor penetration depth - High concentration of contrast agent needed (10^3^ - 10^6^ ng) - Fluorescent transgenic reporters may be needed | No |
| OEM | Visible to infrared light | ≥ 1 mm | ≤ 1 cm | - High sensitivity and specificity - Real-time imaging | - Limited penetration depth - Higher concentration of contrast agent needed (10^3^ - 10^6^ ng) | Yes |
| MRI | Radiofrequency waves | 0.01 – 0.1 mm (small animal MRI);  0.5 - 1.5 mm (clinical MRI) | No limit | - High spatial resolution - Superior soft tissue contrast - Non-contrast techniques available - Whole body and anatomical imaging | - Lower contrast sensitivity than PET and SPECT - Higher concentration of contrast agent needed (10^3^ - 10^6^ ng) | Yes |
| SPECT | Gamma rays | 0.5 – 2 mm (microSPECT);  7 – 15 mm (clinical SPECT) | No limit | - High sensitivity. - Only a trace amount of contrast agent needed (1 - 100 ng) - Whole body imaging | - Exposure to ionizing radiation | Yes |
| PET | Annihilation photons | 1 – 2 mm (microPET);  6 – 10 mm (clinical PET) | No limit | - High sensitivity - Only a trace amount of contrast agent needed (1 - 100 ng) - Whole body imaging | - Exposure to ionizing radiation | Yes |

*IVM = intravital microscopy; OEM = optical endomicroscopy; MRI = magnetic resonance imaging; SPECT = single photon emission computed tomography; PET = positron emission tomography.*
